# BRD4770 inhibits vascular smooth muscle cell proliferation via SUV39H2, but not EHMT2 to protect against neointima formation

**DOI:** 10.1007/s13577-023-00924-4

**Published:** 2023-06-12

**Authors:** Tai-Qiang Chen, Xian Guo, Bo Huo, Xiao-Xuan Zhong, Qun-Hui Wang, Yue Chen, Xue-Hai Zhu, Gao-Ke Feng, Ding-Sheng Jiang, Ze-Min Fang, Xiang Wei

**Affiliations:** 1 Division of Cardiothoracic and Vascular Surgery, Sino-Swiss Heart-Lung Transplantation Institute, Tongji Hospital, Tongji Medical College, Huazhong University of Science and Technology, Wuhan, China; 2 Key Laboratory of Organ Transplantation, NHC Key Laboratory of Organ Transplantation, Key Laboratory of Organ Transplantation, Minist of Education, Chinese Academy of Medical Sciences, Wuhan, China; 3 Department of Cardiology, Renmin Hospital of Wuhan University, Wuhan, China

**Keywords:** Vascular intimal hyperplasia, Vascular smooth muscle cell, Proliferation, BRD4770, G2/M phase, SUV39H2

## Abstract

The behavior of vascular smooth muscle cells (VSMCs) contributes to the formation of neointima. We previously found that EHMT2 suppressed autophagy activation in VSMCs. BRD4770, an inhibitor of EHMT2/G9a, plays a critical role in several kinds of cancers. However, whether and how BRD4770 regulates the behavior of VSMCs remain unknown. In this study, we evaluate the cellular effect of BRD4770 on VSMCs by series of experiments in vivo and ex vivo. We demonstrated that BRD4770 inhibited VSMCs’ growth by blockage in G2/M phase in VSMCs. Moreover, our results demonstrated that the inhibition of proliferation was independent on autophagy or EHMT2 suppression which we previous reported. Mechanistically, BRD4770 exhibited an off-target effect from EHMT2 and our further study reveal that the proliferation inhibitory effect by BRD4770 was associated with suppressing on SUV39H2/KTM1B. In vivo, BRD4770 was also verified to rescue VIH. Thus, BRD4770 function as a crucial negative regulator of VSMC proliferation via SUV39H2 and G2/M cell cycle arrest and BRD4770 could be a molecule for the therapy of vascular restenosis.

**Supplementary Information:**

The online version contains supplementary material available at 10.1007/s13577-023-00924-4.

## Introduction

Coronary artery disease (CAD), with the pathology of coronary artery stenosis, spasm, or embolism, was considered to be one of the major causes of mortality and morbidity all over the world. It was estimated that one-third adults in USA were suffering from cardiovascular diseases, including > 17 million with CAD and 10 million with angina pectoris [1]. Nowadays, angioplasty and vascular grafting were highly effective in CAD treatments. However, the long-term outcome of postoperative patients was less optimistic due to the lumen restenosis. It was found that the restenosis occurred with the frequency ranging from 25 to 50% in patients treated with percutaneous coronary intervention, and worse still, the majority of which required further myocardial revascularization procedures within 6 months [2]. As reported, neointimal hyperplasia after stent implantation is one key process of restenosis, in which foreign stents impels endothelial cells release a series of cytokines and growth factors, inducing abnormal proliferation and migration of VSMC to form lumen reduction [3]. In the cases of patients treated with saphenous vein grafts (SVG), restenosis rate was approximately up to 25% within the first 12 to 18 months after surgery [4]. It was verified that intimal hyperplasia is main reason for pathophysiology of failure. Under direct or indirect stimulation by multiple cytokines, the phenotype of SMCs switches from quiescent contractile state to synthetic state in SVG, that results excessive proliferation and intimal migration [5]. Accumulating study showed that the pathophysiology of restenosis was related to procedure of vascular intimal hyperplasia (VIH). The excessive proliferation and migration of vascular smooth muscle cells (VSMCs) greatly contributed VIH after angioplasty or vascular grafting surgery [6]. Therefore, the regulating behavior of VSMCs and blocking process of VIH were important for the further treatment of restenosis.

Given to the fact that epigenetics was related to variety of multiple gene expression regulation, the role of epigenetics in pathophysiology of VIH was getting much more attention in CAD and the investigation of epigenetics in VIH development was meaningful for clinical therapeutics. Moreover, the epigenetic regulation of VSMCs, to inhibit excessive proliferation and migration, may also be favorable for the prevention of VIH. It was reported that vascular injury elevated the expression of EZH2 and the downstream H3K27, which promoted VSMC proliferation and migration in final [7]. Withal, another research revealed that epigenetic regulator UHRF1 could trigger VSMCs proliferation by directly repressing promoters of cell-cycle inhibitor genes via the methylation of DNA and histones, and further animal experiment verified that UHRF1 deletion prevented intimal hyperplasia [8]. Thus, epigenetic regulation was a vital process in VSMCs proliferation and migration for the formation of VIH, and more epigenetic regulators might be involved, which remains to be explored.

EHMT2 (also known as G9a), a Su (var), enhancer of Zeste was a trithorax (SET) domain-containing protein with histone lysine methyltransferase activity. It was known that EHMT2 was responsible for the monomethylation and dimethylation of lysine 9 on histone H3 (H3K9me1, H3K9me2) [9]. Intriguingly, the methylation of lysine 9 on histone H3 (H3K9) was often found in the promoter regions of aberrantly silenced tumor suppressor genes in cancer cells. As a result, the EHMT2 was considered to be associated with transcriptional silencing [10]. Moreover, the methylation of H3K9 and gene transcriptional silencing induced by EHMT2 might also be related to cell proliferation [11], cell differentiation [9], cell apoptosis [12], cell autophagy [13] and so on. As mentioned in our previous work, the inhibition of EHMT2 by BIX01294 accelerated the formation of autophagosomes via promoting SQSTM1/p62 and BECN1/beclin1 expression in VSMCs [14]. However, whether the regulation of EHMT2 could affect VIH was unknown.

BRD4770, besides BIX-01294, was reported to be another compound which selectively inhibited EHMT2. It reduced cellular levels of mono- and dimethylated H3K9 through EHMT2 [15, 16]. The suppression of EHMT2 by BRD4770 was suggested to be similar with the knockdown of EHMT2 [16]. What’s more, BRD4770 was verified not affect cell viability even in high concentration [15]. Thus, it might be a meaningful compound to regulate the function of EHMT2. At present, whether BRD4770 regulated behavior of VSMCs and whether BRD4770 regulated autophagy through EHMT2 inhibition remained unknown. In addition, whether BRD4770 also altered VIH still needed to be further explored.

In the present work, we found that BRD4770 inhibited VSMCs proliferation via blocking cell cycle in G2/M phase instead of inducing autophagy through EHMT2. The inhibitory effect was mediated by affecting the expression of suppressor of variegation 3–9 homolog2 (SUV39H2). Meanwhile, it was also demonstrated that BRD4770 suppressed VIH in vivo, which suggested  BRD4770 was potentially and effective epigenetic related compound to inhibit restenosis.

## Materials and methods

### Vascular smooth muscle cells culture and treatment

Primary human aortic vascular smooth muscle cells (p-HAVSMC) were cultured from human ascending aortic tissues of five healthy DBD donors. All written informed consents were signed by their immediate family. Human ascending aortas were obtained from heart transplantation at Tongji Hospital, Tongji Medical College, Huazhong University of Science and Technology. As described before [14], aortic tissues were transferred into new petri dish after the removal of blood and connective tissue on the surface of vessels. Intimal and adventitial tissues were stripped with forceps under the stereo microscope. Medial layer of vessels was then cut into small pieces and transferred to cell-culture flasks. The tissue blocks were spread on the bottom of flask with appropriate interval of approximately 2 mm, and then 5 mL of DME/F12 medium supplemented with 10% FBS, 1% l-glutamine, and antibiotics were added to the flask, and next the lid was loosely screwed on. The flask was transferred to incubator and kept upright for 30 min to ensure tissue blocks attach closely to the flask. Then, the flask was lowered and not moved for 5 days until long spindle-shaped smooth muscle cells were observed around the blocks. The medium was renewed every 3 days and the state of cells was observed closely. Cells were passaged when degree of cell fusion reached approximately 80% and distributed evenly. Given healthy p-HAVSMCs, passages 3–5 were used to perform experiments. Furthermore, p-HAVSMCs isolated from the ascending aortas of heart transplantation donors have been identified by SMC marker α-SMA [14]. The rabbit aortic vascular smooth muscle cells (RAVSMCs) were cultured with DME/F12 medium (SH30023; Hyclone) mixed with 10% fetal bovine serum (1,767,839; Thermo Fisher Scientific) and 1% penicillin–streptomycin (15,140–122; Thermo Fisher Scientific). RAVSMCs and p-HAVSMCs were treated with BRD4770 (S7591; Selleck) at different concentration as indicted after starved for 12 h. Cells were infected with lenti-mCherry-GFP-LC3, lenti-shRNA, lenti-shSQSTM1, lenti-shBECN1, lenti-flag, lenti-EHMT2, lenti-SRF, lenti-HDAC1, lenti-HDAC3, lenti-SETD8, lenti-SUV39H1 and lenti-SUV39H2 lentivirus, respectively. After lentivirus infection for 24 h, the medium was changed and cells were prepared for other treatment. The LDH release was detected using Cytotoxicity LDH Kit-WST^®^ kit (CK12; Dojindo). Cell counting was performed by Cellometer Mini (Nexcelom).

### Plasmids

The EHMT2/G9a, KMT5a/Setd8 overexpression plasmids were constructed into Phage-flag plasmids at MluI and SalI restriction enzyme sites. Histone deacetylase 1(HDAC1), histone deacetylase 3(HDAC3), suppressor of variegation 3–9 homolog 1(SUV39H1), SUV39H2, and serum response factor (SRF) overexpression plasmids were constructed into Phage-flag plasmids at MluI and XhoI restriction enzyme sites. Double-strand oligonucleotides of shRNA targeting to SQSTM1 and BECN1 were cloned into pLKO.1 plasmid at AgeI and EcoRI restriction enzyme sites. Primer sequences were provided in Supplementary Table S1.

### Western blot

Western blot was performed as previously reported [17]. Proteins from p-HAVSMCs and RAVSMCs were isolated by RIPA and detected on SDS-PAGE gel. After proteins transferred to PVDF membranes by electrophoretic transfer, the membranes were blocked with 5% non-fat milk for 1 h at room temperature, then incubated with indicated primary antibody overnight at 4 °C. After incubating with the peroxidase-conjugated secondary antibody at room temperature for 2 h, the protein signals were detected using the ChemiDocTM XRS + system (Bio-Rad). Total protein shown by stain-free gels served as a loading control. Primary antibodies used in this study are provided in Supplementary Table S2.

### Real-time PCR

Real-time PCR was performed as previously reported [17]. To recapitulate, cellular total mRNA was extracted using TRI Reagent^®^ Solution (AM9738; ThermoFisher Scientific). Next, the extracted mRNA was reversely transcribed into cDNA using a transcriptor first strand cDNA synthesis kit (4,896,866,001; Roche). The relative mRNA levels of targets were detected by CFX connect™ real-time PCR detection system (Bio-Rad) using iQ™SYBR^®^ green supermix (1,708,884; Bio-Rad). Primers used in this study were shown in Supplementary Table S3.

### Immunofluorescence analysis

Immunofluorescence staining was performed as reported [14]. Briefly, the cultured RAVSMCs and p-HAVSMCs were stained with Ki67 (ab16667, Abcam) antibody. Secondary antibody was incubated for 60 min and followed with DAPI staining. Images were taken by Olympus light microscope BX53 system. RAVSMCs infected with lenti-mCherry-GFP-LC3 were fixed with 4% paraformaldehyde for 15 min in darkness. After that, cells were taken to capture image by Olympus light microscope BX53 system directly.

### Cell proliferation measurement using EdU incorporation assay

EdU incorporation assay was performed by Cell-Light™ Edu Apollo567 In Vitro kit (C10310-1, RIBOBIO). First, RAVSMCs and p-HAVSMCs were planted in 24-well plates at 1 × 10^4^ cells per well. The cells were then treated with 5 μM of BRD4770 or DMSO for 24 h, and incubated with 50 μM EdU medium for 2 h. Following fixed with 4% paraformaldehyde and incubated with 2 mg/ml glycine, the cells were incubated with 0.5% Triton X-100 and 1 × Apollo staining solution for 30 min. Subsequently, 0.5% Triton X-100 PBS solution was added again and cells were incubated with 1 × Hoechst 33,342 for 30 min at room temperature. Finally, cells were observed by fluorescence microscopy.

### Flow cytometry

RAVSMCs and p-HAVSMCs numbered about 2–5 × 10^6^ were collected after digestion of trypsin. Cells were washed by cold PBS, centrifuged in 1000 r/min for 10 min, resuspended in 500 μL PBS and added 5 mL cold 70% ethanol to fixation at 4 ℃ overnight. Then cold 70% ethanol was discarded and the cells were washed with cold PBS in twice. Subsequently, cells were resuspended by 150 μL RNase, and 150 mL PI (P4864, Sigma-Aldrich) was added to the system. After staining for 2 h in darkness, cell cycle was detected by BD FACS Aria™ III Sorter.

### Carotid artery wire injury model

All animal protocols were approved by the Animal Care and Use Committee of Tongji Hospital at Huazhong University of Science and Technology, China (approval number: TJH-202209008). All animal experiments were carried out according to Guide for the Care and Use of Laboratory Animals from the National Research Council (US) Institute for Laboratory Animal Research. Fourteen male mice (C57BL/6 background) aged 6 to 8 weeks were randomly divided into two groups that eight for DMSO and six for BRD4770. All mice were housed in a ventilated room at a stable condition (50–60% humidity and 25 °C) with a 12 h light/dark cycle. Mice were allowed to obtain a standard diet and water. Carotid artery wire injury surgery was performed as described previously [18]. After the surgery, the mice were treated through an intraperitoneal injection of BRD4770 in a dose of 1 mg/kg/d, while the mice in control group were treated with the same dose of DMSO. The animal tissues were collected at 28th day after surgery for histological analysis. All male C57BL/6 mice were purchased from Tongji Medical University (Experimental Animals Center of Tongji, China) and housed in a ventilated room at a stable condition (24 °C and 50–60% humidity) with a 12 h light/dark cycle. Mice were allowed to free obtain a standard diet and water.

### Histological analysis

At 28th day after surgery, the mice were killed through the intraperitoneal injection of an overdose of sodium pentobarbital (150 mg/kg). As reported previously[18], the carotid arteries were harvested after circulation perfusion and fixed with 4% paraformaldehyde dissolved in PBS. The tissues were further formalin-fixed and embedded with paraffin. Serial cross-Sects. (5 μm) were produced from the entire region at the bifurcation site of the left carotid artery. For histological analysis, the sections were stained haematoxylin eosin after deparaffinization and rehydration. Tissues harvested from injured side belonged to injured group and non-injured side for sham group. Three slices were examined each group followed by average number calculated. Images were taken by microscope and the level of neointima formation was determined based on intimal area and I/M ratios which calculated by Image-Pro Plus 6.0.

### RNA sequencing and transcriptomic analysis

RAVSMCs was treated with BRD4770 5 μM or DMSO for 24 h. Then, respective total RNAs were extracted and random primers were used to compose cDNA. Sequencing was carried out using Illumina platform with sequencing strategy PE150 (Anoroda Genome, Beijing, China). After the data preprocessing, fragments per kilobase of exon per million fragments mapped (FPKM) and differentially expressed genes (DEGs) were then calculated. Genes satisfying |log2 Fold change|≥ 1 and padj < 0.05 were considered to be significant differentially expressed genes. Kyoto Encyclopedia of Genes and Genomes (KEGG) tool was used for pathway analysis of the DEGs.

### Statistical analysis

All the data were represented as mean ± standard deviation (SD) in the present study. Student’s two-tailed t-test was used to compare the means of two groups. Multiple groups comparisons were achieved by using one-way ANOVA test with least significant difference (equal variances assumed) or Tamhane T2 (equal variances not assumed) in SPSS software (version 13.0). *p* < 0.05 is considered as statistical significance.

## Results

### BRD4770 inhibited the expression of H3K9me1/2 and induced the cell-cycle blockage of G2/M phase in RAVSMCs

**Fig. 1:**
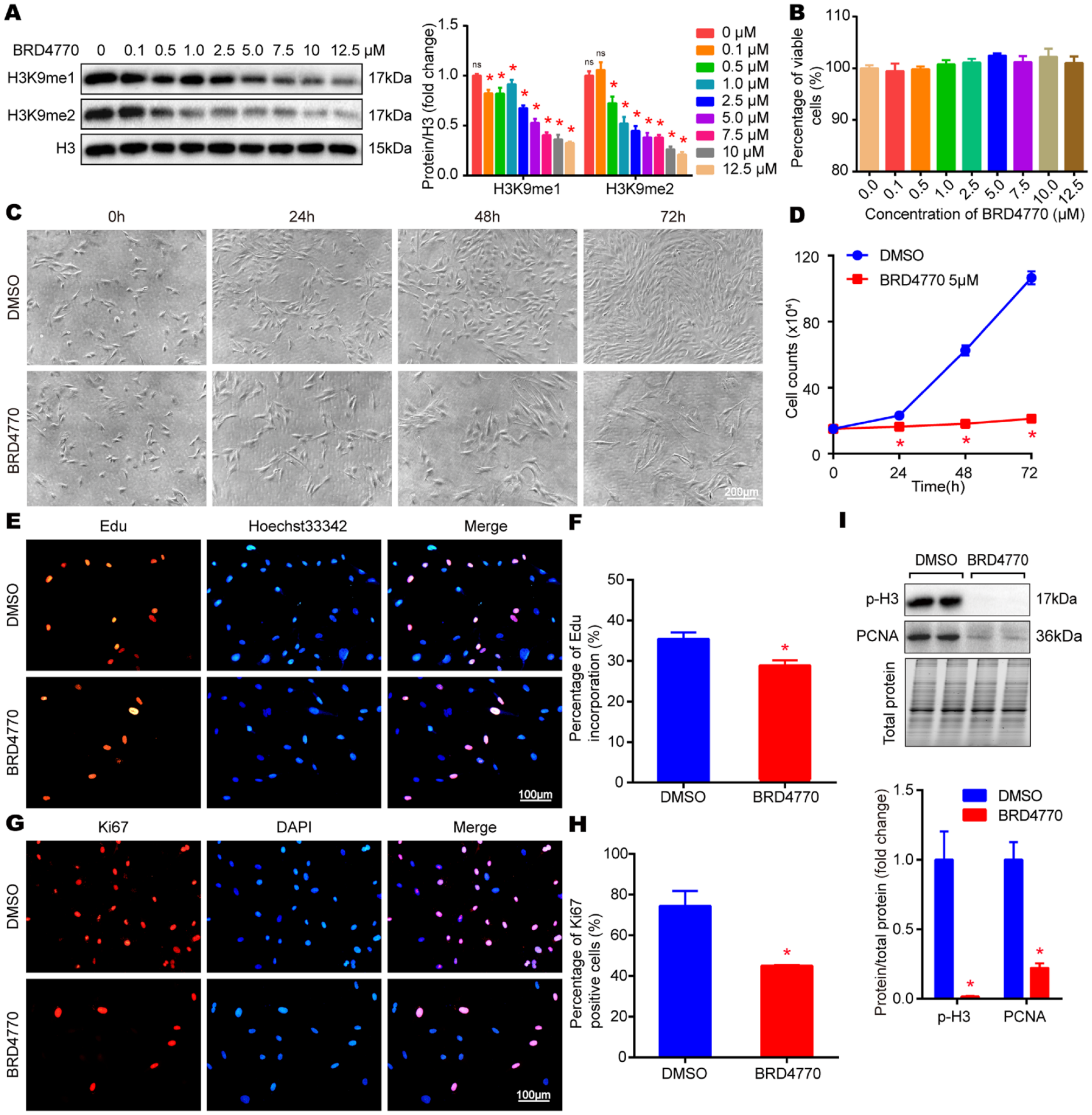
BRD4770 inhibited the expression of H3K9me1/2 and proliferation of RAVSMCs. **A** Western blots results and relative protein quantification of H3K9me1, H3K9me2 in RAVSMCs, and protein H3 served as a loading control, *p < 0.05, ns means no statistical sense. **B** The percentage of viability was evaluated by LDH kit after BRD4770 treatments with indicated doses for 24 h (*n* = 3), *p < 0.05. **C** Representative images of RAVSMCs treated with DMSO or BRD4770 5 μM for indicated times (*n* = 3). **D** Growth curve of RAVSMCs treated with BRD4770 5 μM or DMSO at different time point (*n* = 3), *p < 0.05. **E** Representative immunofluorescence staining of Edu after treated with DMSO or BRD4770 5 μM for 24 h in RAVSMCs. Red points for Edu staining nucleus and blue points for nucleus of live cells. **F** Edu incorporation rate of RAVSMCs treated with DMSO or BRD4770 5 μM for 24 h (*n* = 3), *p < 0.05. **G** Representative immunofluorescence staining of Ki67 after treated with DMSO or BRD4770 5 μM for 24 h in RAVSMCs. Red points for Ki67 positive nucleus and blue points for nucleus of live cells.** H** Percentage of Ki67 positive RAVSMCs after treated with DMSO or BRD4770 5 μM for 24 h (*n* = 3), *p < 0.05. **I** Western blots results and relative protein quantification of p-H3, PCNA after treatment of BRD4770 5 μM or DMSO for 48 h in RAVSMCs, total protein served as a loading control (*n* = 4), *p < 0.05

**Fig. 2:**
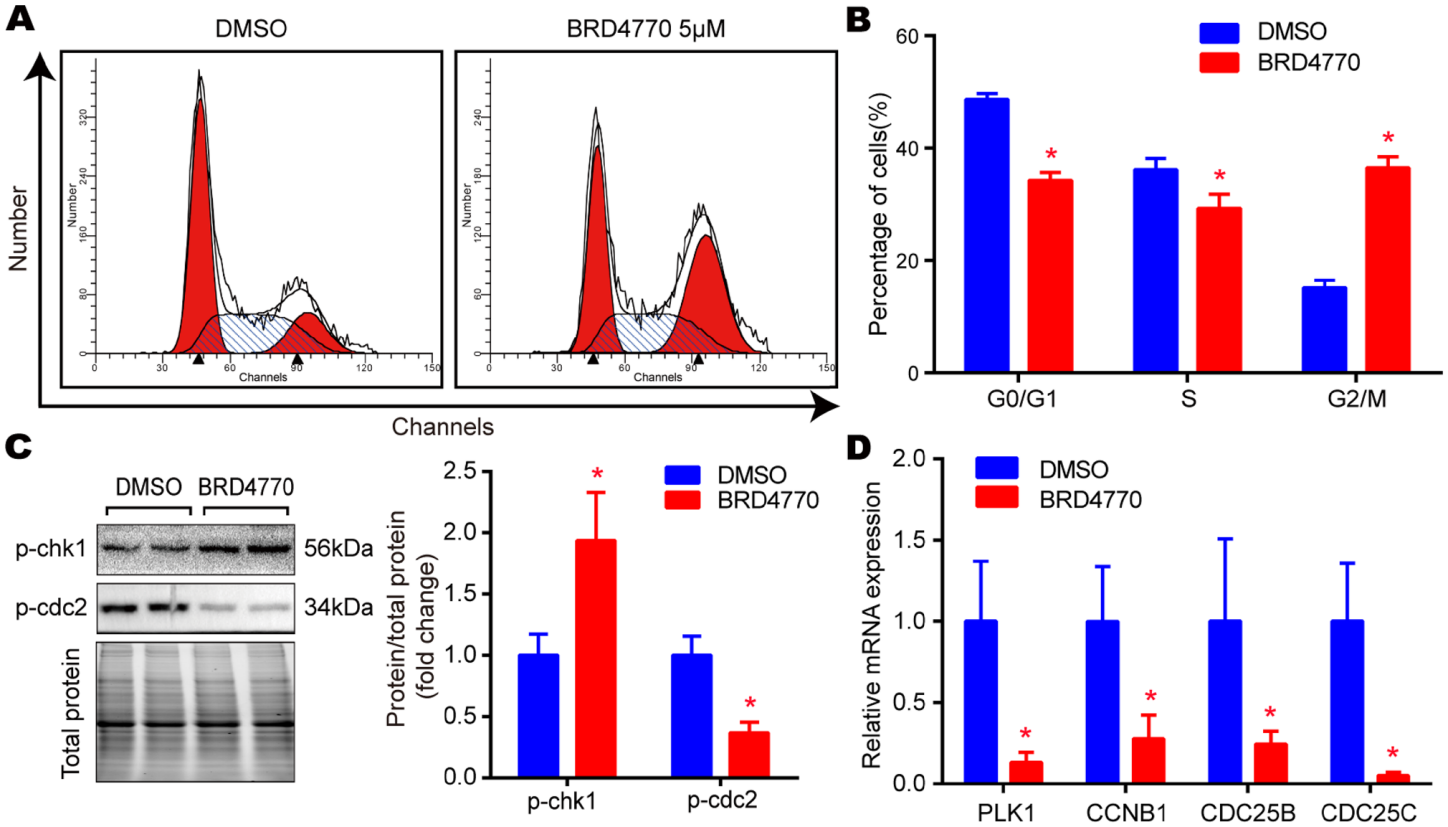
BRD4770 induced G2/M phase blockage in RAVSMCs. **A** Flow cytometry of cell cycle after treatment of BRD4770 5 μM or DMSO for 48 h in RAVSMCs. **B** Histograms of the cell percentages in the different phases of cell cycle after treatment of BRD4770 5 μM or DMSO for 48 h in RAVSMCs (*n* = 3), *p < 0.05. **C** The western blots results and relative protein quantification of p-chk1 and p-cdc2 after treatment of BRD4770 5 μM or DMSO for 48 h in RAVSMCs, and total protein served as a loading control (*n* = 4), *p < 0.05.** D** the mRNA expression of PLK1, CCNB1, CDC25B and CDC25C after treatment of BRD4770 5 μM or DMSO for 48 h in RAVSMCs (*n* = 4), *p < 0.05

To explore the effect of BRD4770 on RAVSMCs, RAVSMCs were treated with increasing concentrations of BRD4770 for 24 h after serum starvation. The result indicated that BRD4770 concentration-dependently repressed H3K9me1/2 level in RAVSMCs. BRD4770 in 5 μM was recognized sufficient concentration, since the H3K9me1/2 inhibitory efficiency was ≥ 50% (Fig. 1A). Furthermore, cell injury assay demonstrated that BRD4770 treatment from 0.1 μM to 12.5 μM did not exhibit cytotoxicity to RAVSMCs. Suggesting that BRD4770 was non-cytotoxic in its effective dosage (Fig. 1B). Therefore, 5 μM BRD4770 was chosen for the following in vitro experiments. Notably, 5 μM BRD4770 treatment resulted in a significantly reduced number of RAVSMCs at 48 h and 72 h with the evidence of cell growth curve accompanied with enlarged and flattened cell morphology (Fig. 1C and D). In Edu incorporation assay, BRD4770 treatment decreased the number of Edu positive cells in RAVSMCs (Fig. 1E and F). Moreover, Ki67 marked immunofluorescence test revealed that proliferative Ki67 positive cells apparently decreased after BRD4770 treatment in RAVSMCs (Fig. 1G and H). Consistently, the expression of proliferative-marker like p-H3, PCNA was also blocked after treatment of BRD4770 in RAVSMCs (Fig. 1I). Cell cycle was then evaluated by flow cytometry. Results showed that more cells were trapped in the G2/M phase while fewer cells in both G0/G1 phase and S phase after BRD4770 treated (Fig. 2A and B). Meanwhile, BRD4770 treatment also showed impacts on the expression of G2/M phase related proteins, including that cell cycle progression negatively regulated protein, phosphorylated checkpoint kinase1(p-chk1) was up regulated, and the G2 phase key protein, phosphorylated cell division control protein 2 homolog (p-cdc2) was down regulated (Fig. 2C). Likewise, it was found that the mRNA level of G2/M phase key genes such as polo like kinase 1 (PLK1), cyclin B1 (CCNB1), cell division cycle 25B(CDC25B) and cell division cycle 25C(CDC25C) all decreased after BRD4770 treated (Fig. 2D). To sum up, BRD4770 concentration-dependently inhibited the expression of H3K9me1/2 and inhibited the proliferation of RAVSMCs via blocking cell cycle in G2/M phase.

### BRD4770 inhibited the proliferation and blocked G2/M phase in primary human aortic vascular smooth muscle cells

**Fig. 3:**
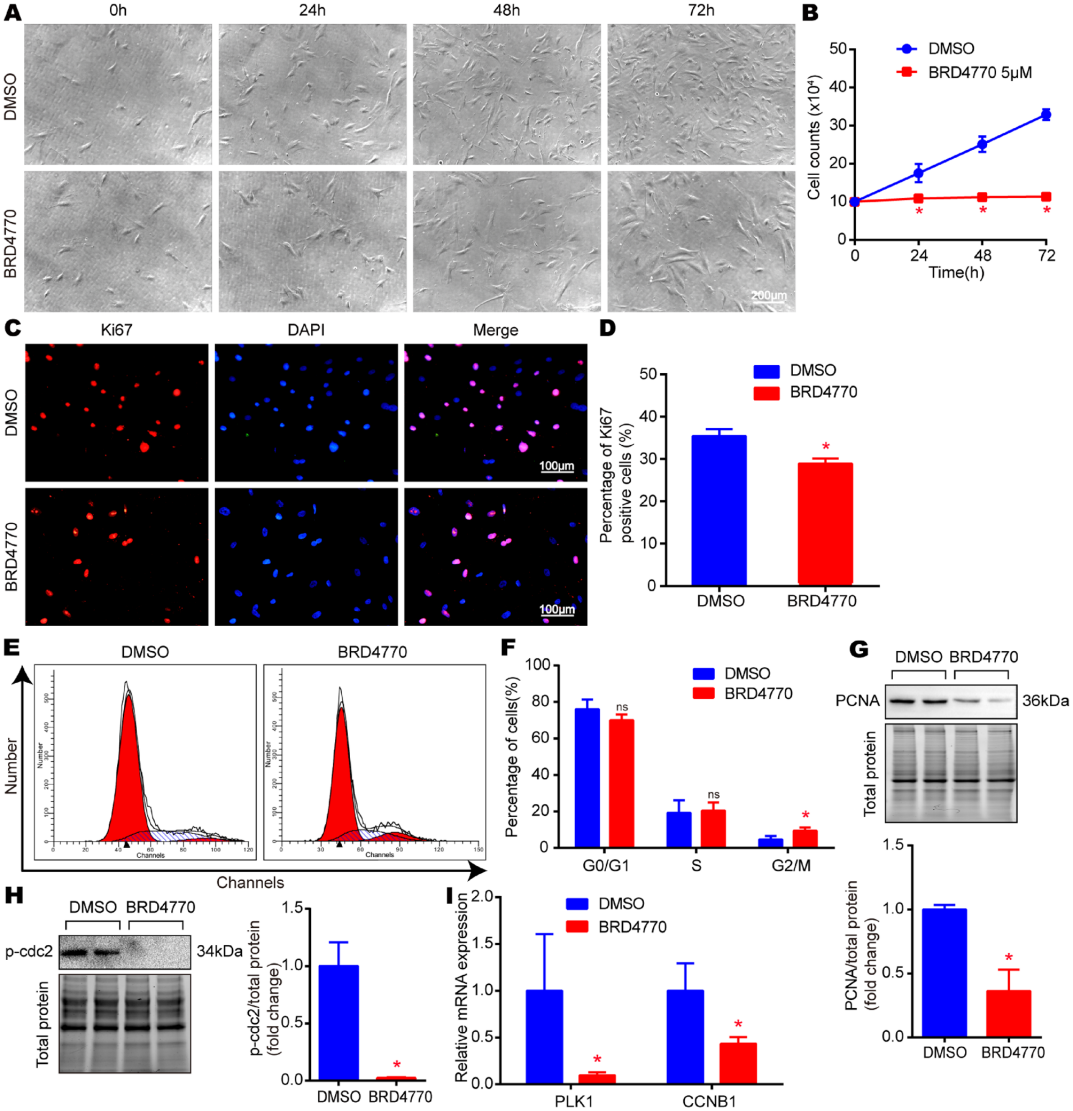
BRD4770 inhibited proliferation and blocked G2/M phase as well in p-HAVSMCs. **A** Representative images of p-HAVSMCs treated with DMSO or BRD4770 5 μM for indicated times (*n* = 3). **B** Growth curve of p-HAVSMCs treated with BRD4770 5 μM or DMSO at different time point (*n* = 3), *p < 0.05. **C** Representative immunofluorescence staining of Ki67 after treated with DMSO or BRD4770 5 μM for 24 h in p-HAVSMCs. **D** Percentage of Ki67 positive cells in p-HAVSMCs treated with DMSO or BRD4770 5 μM for 24 h (*n* = 3), *p < 0.05. **E**. Flow cytometry of cell cycle after treatment of BRD4770 5 μM or DMSO for 48 h in p-HAVSMCs. **F** Histograms showing the percentages of cells in the different phases of cell cycle after treatment of BRD4770 5 μM or DMSO for 48 h in p-HAVSMCs (*n* = 3), *p < 0.05. **G** Western blots results and relative protein quantification of PCNA after treatment of BRD4770 5 μM or DMSO for 48 h in p-HAVSMCs, and total protein served as a loading control (*n* = 4), *p < 0.05. **H** Western blots results and relative protein quantification of p-cdc2 after treatment of BRD4770 5 μM or DMSO for 48 h in p-HAVSMCs, and total protein served as a loading control (*n* = 4), *p < 0.05.** I** Relative mRNA expression of PLK1 and CCNB1 after treatment of BRD4770 5 μM or DMSO for 48 h in p-HAVSMCs (*n* = 4), *p < 0.05

For further verified the effect of BRD4770 in VSMCs, p-HAVSMCs were treated with 5 μM BRD4770 for indicated times. The result revealed that the cell proliferation was blocked by BRD4770 (Fig. 3A and B). In addition, the number of proliferative Ki67 positive cells decreased after BRD4770 treatment in p-HAVSMCs (Fig. 3C and D). In the test of flow cytometry, more p-HAVSMCs were trapped in the G2/M phase after BRD4770 treated (Fig. 3E and F). Similarly, BRD4770 was also shown to block the expression of PCNA and p-cdc2 in p-HAVSMCs (Fig. 3G and H). At the mRNA level, the expression of PLK1 and CCNB1 was inhibited after BRD4770 incubation (Fig. 3I). Totally, results indicated that BRD4770 inhibited the proliferation and induced G2/M cell cycle arrest in p-HAVSMCs, which were in consistent with the results acquired in RAVSMCs.

### BRD4770 showed no effect on autophagy, and the overexpression of EHMT2, SQSTM1 or BECN1 didn't rescue the number of VSMCs suppressed by BRD4770

**Fig. 4:**
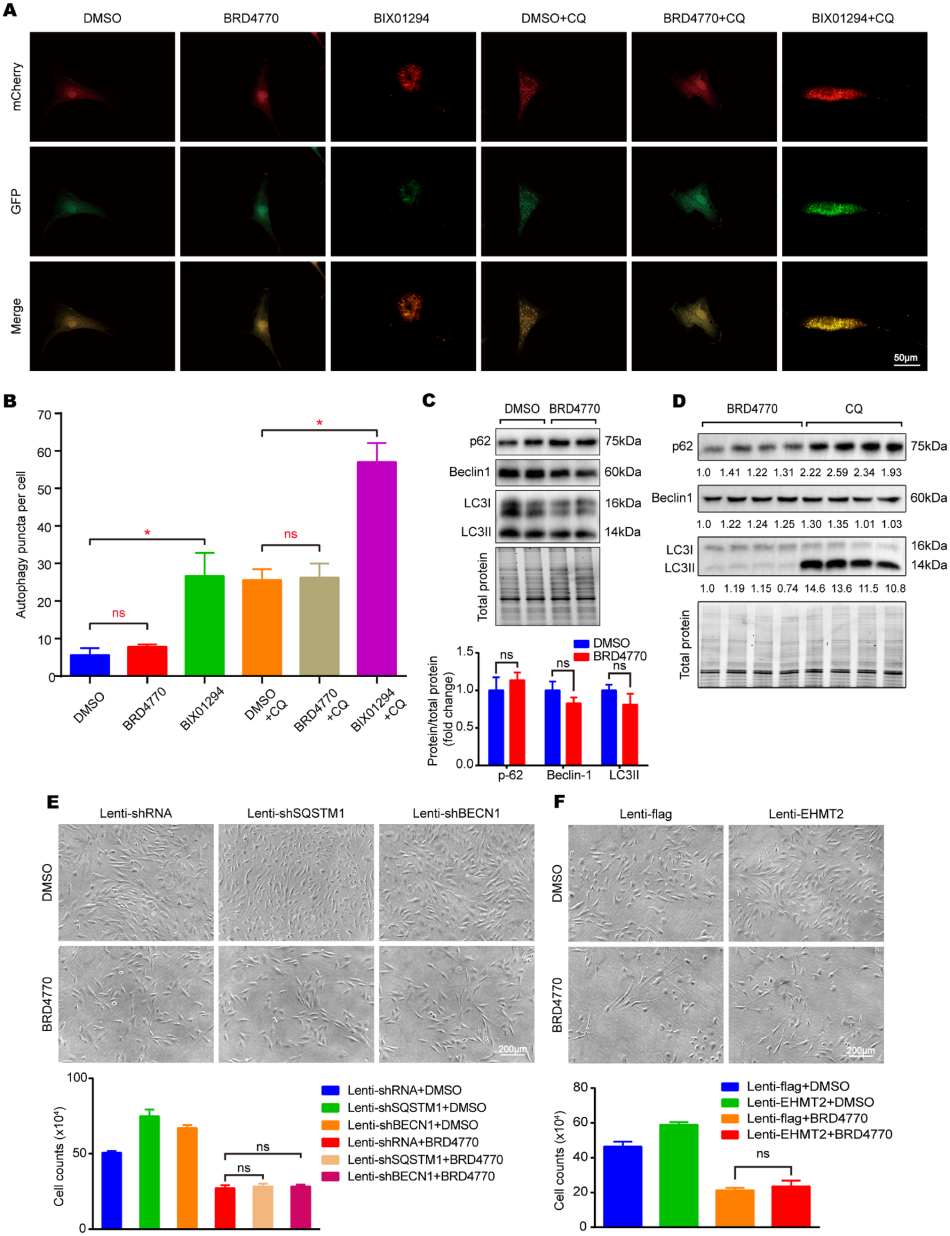
The effect of BRD4770 on VSMCs is independent of autophagy or G9a. **A** Representative immunofluorescence images of RAVSMCs infected with lenti-mCherry-GFP-LC3 after treated with BRD4770 5 μM, BIX01294 5 μM, DMSO, BRD4770 5 μM + CQ, BIX01294 5 μM + CQ and DMSO + CQ for 8 h. In the merged image, yellow puncta indicated autophagosomes and the puncta with GFP quenched and red puncta remaining indicated autolysosomes that have fused with lysosomes in RAVSMCs. DMSO served as negative control and BIX01294 served as positive control (*n* = 3), *p < 0.05. **B** Autophagy puncta per cell calculated in RAVSMCs infected with lenti-mCherry-GFP-LC3 with indicated stimulations for 8 h, DMSO served as negative control, *p < 0.05. **C** Western blots results and relative protein quantification of p-62, beclin-1, LC3II treated with BRD4770 5 μM or DMSO for 48 h in RAVSMCs, and total protein served as a loading control (*n* = 4). **D** Western blots results and relative protein quantification of p-62, beclin-1, LC3II treated with BRD4770 5 μM or CQ alone for 48 h in RAVSMCs, and total protein served as a loading control (*n* = 4). **E** Cell images and histogram of RAVSMCs infected with lenti-shRNA, lenti-shSQSTM1 and lenti-shBECN1, and then treated with BRD4770 5 μM or DMSO (*n* = 3) for 48 h. **F** Cell images and histogram of RAVSMCs infected with lenti-EHMT2, lenti-flag, and then treated with BRD4770 5 μM or DMSO (*n* = 3) for 48 h. Mark “ns” means no statistical sense

Our previous study demonstrated that EHMT2 inhibits VSMCs autophagy via reducing SQSTM1/p-62 and BECN1/beclin-1 expression [14]. As previously stated, BRD4770 was an effective inhibitor of EHMT2 [16]. However, whether BRD4770 affected EHMT2 related autophagy to inhibit VSMCs growth was unknown. To confirm the relation between BRD4770 and autophagy, a mCherry-EGFP-LC3 reporter study was conducted. Surprisingly, stimulation of RAVSMCs with BRD4770 did not increase the number of autophagy puncta. Instead, BIX01294, another inhibitor of EHMT2, induced an increasing of autophagy puncta as reported before [14] (Fig. 4A and B). Furthermore, chloroquine (CQ, blocking degradation of autophagosome components) was used to test role of BRD4770 in autophagic flux. CQ treatment resulted in LC3II accumulation in RAVSMCs. In our study, there was no significant difference after BRD4770 added (Fig. 4A and B). The expression of autophagy related markers including p62, beclin1 and LC3I/II were detected by western blot, and no remarkable changes were showed upon BRD4770 treatment (Fig. 4C). Besides that, protein p62 and LC3II increased in CQ group compared to BRD4770 group (Fig. 4D). Furthermore, we found that the knockdown of p62 or beclin1 didn't rescue cell numbers reduced by BRD4770 in RAVSMCs (Fig. 4E). Cell number suppressed by BRD4770 didn't increase though we constructed EHMT2 over-expressed cell line (Fig. 4F). In summary, results showed that BRD4770 didn't influence the autophagic flux, neither autophagic activation nor degradation of autophagosome. And the inhibition of cell number by BRD4770 was irrelevant with EHMT2 or its related autophagy.

### BRD4770 influenced cell cycle pathway and cell division as demonstrated by transcriptomic analysis

**Fig. 5:**
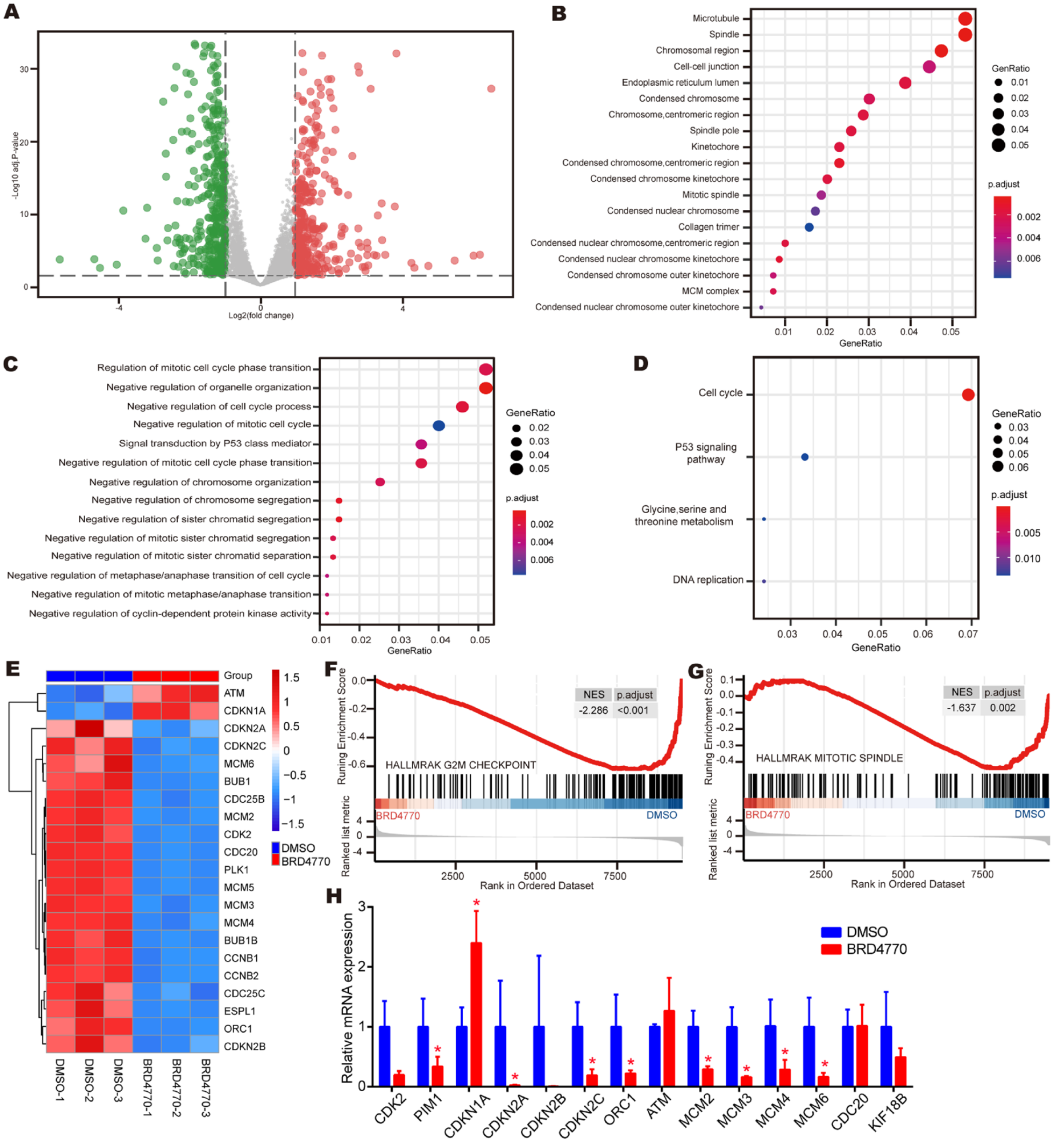
GO & KEGG pathway enrichment analysis of DEGs in RAVSMCs treated with BRD4770 or DMSO for 24 h.** A** Volcano image demonstrates the change in the BRD4770 treatment group compared to the control group (DMSO): red and green dots indicate differentially expressed genes with statistical significance. Red dots indicate upregulation of gene expression (fold change ≥ 2, padj < 0.05), whereas green dots indicate downregulation of gene expression (fold change ≤ −2, padj < 0.05). Gray dots indicated that there was no statistically significant difference in gene expression (|fold change|< 2 or padj >  = 0.05). **B** Cell component (CC) of DEGs. **C** Biological processes (BP) of DEGs. **D** KEGG pathway analyses. **E** Hierarchical clustering dendrogram of gene expression: the horizontal axis at the bottom represents the name of samples and the vertical axis on the right side represents the degree of gene clustering. The vertical axis on the right side represents the name of genes and the horizontal axis at the top represents the degree of clustering of samples. The red color stands for upregulated while the blue color stands for downregulated. It can be concluded that the samples can be divided into clusters generally: the control group of DMSO treatment and the experimental group of BRD4770 5 μM treatment. **F** GSEA which represent gene set enriched of hallmark-G2/M-checkpoint for the two groups of RAVSMCs. **G** GSEA which represent gene set enriched of hallmark- mitotic-spindle for the two groups of RAVSMCs. **H** Relative mRNA expression of CDK2, PIM1, CDKN1A, CDKN2A, CDKN2B, CDKN2C, ORC1, ATM, MCM2, MCM3, MCM4, MCM6, CDC20 and KIF18B after treatment of BRD4770 5 μM or DMSO for 48 h in RAVSMCs (*n* = 4), *p < 0.05

After standardizing the microarray data, 847 statistically significant differentially expressed genes (DEGs) were screened out by the gene coexpression network analysis. The dataset consisted of 430 downregulated genes and 417 upregulated genes between BRD4770 group and DMSO group (Fig. 5A). Results of GO analyses revealed that the cell component (CC) of DEGs primarily had changes in the microtubule, spindle, chromosomal region, cell cell junction and endoplasmic reticulum lumen and so on (Fig. 5B). Biological process (BP) of DEGs had a great enrichment in decreasing gene expression from mitotic cell cycle phase transition, organelle organization, cell cycle process, mitotic cell cycle and P53 class mediator (Fig. 5C). KEGG pathway analyses showed that DEGs mainly enriched in cell cycle, p53 signaling pathway, glycine, serine, threonine metabolism and DNA replication (Fig. 5D). Based on the genome expression patterns, hierarchical clustering was then constructed. The cell cycle related DEGs were listed according to the size of difference. From the horizontal axis at the bottom, it could be concluded that most of cell cycle related genes were downregulated in BRD4770 subgroups (Fig. 5E). The expression of Hallmark gene sets was further examined by gene set enrichment analysis (GSEA) method. The results indicated that G2/M checkpoint related genes were downregulated with BRD4770 treated (Fig. 5F), and the expression of mitotic-spindle genes was also downregulated by BRD4770 (Fig. 5G). Furthermore, on RT-PCR test, it was found that the gene expression of markers related with cell cycle were mostly downregulated after BRD4770 treatment (Fig. 5H). All in all, BRD4770 induced changes of cell cycle related gene expression, which result in changing in cell division and cell proliferation.

### Neither SRF nor HDAC1, HDAC3, HDAC4 and HDAC6 was involved in the inhibitory effect of BRD4770 against VSMCs proliferation

**Fig. 6:**
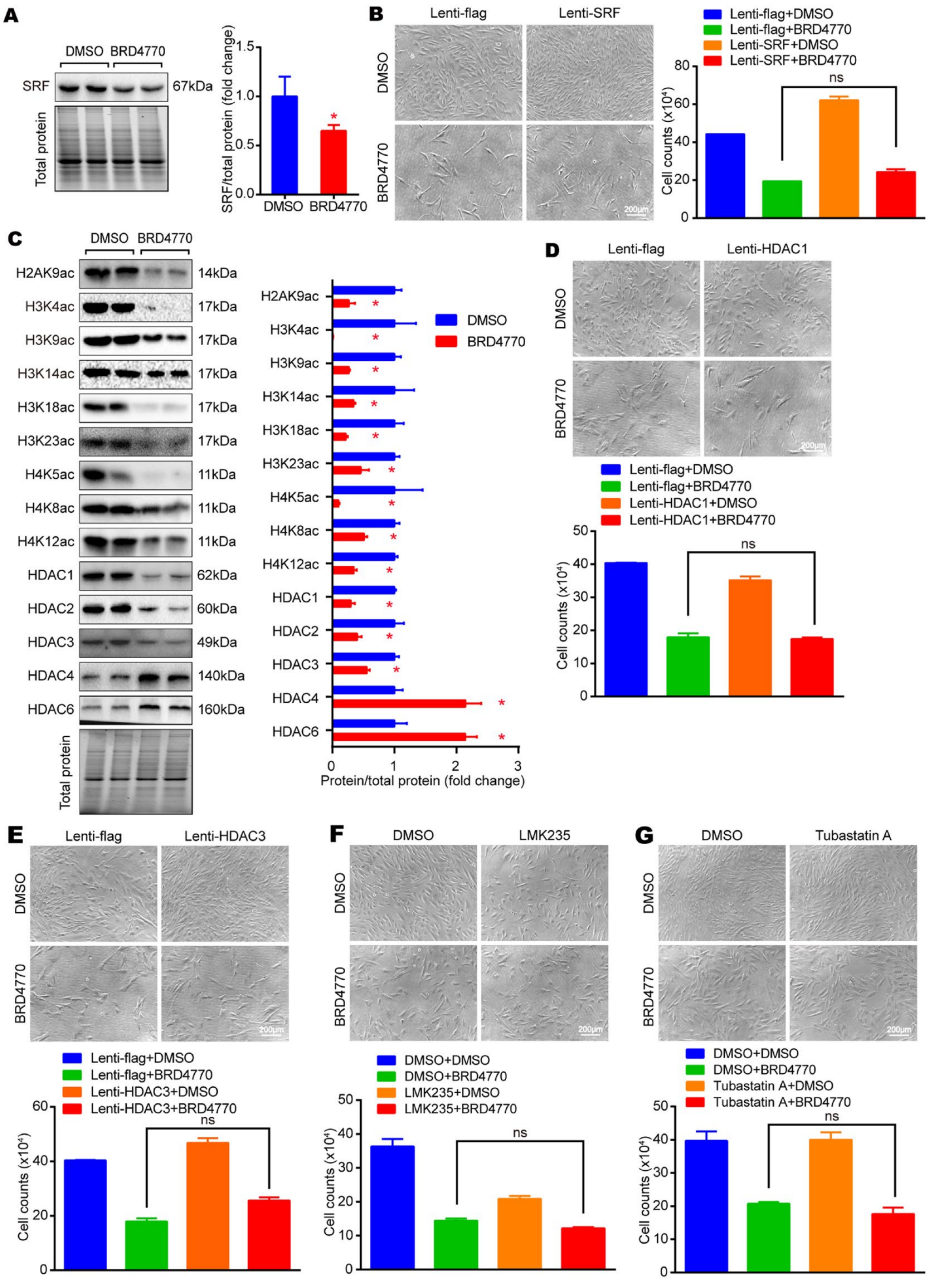
BRD4770 inhibits proliferation of VSMCs not via SRF nor HDACs. **A** Western blots results and relative protein quantification of SRF after treated by BRD4770 5 μM or DMSO for 48 h in RAVSMCs, and total protein served as a loading control (*n* = 4), *p < 0.05. **B** Cell images and histogram of RAVSMCs infected with lenti-SRF, lenti-flag, and then treated with BRD4770 5 μM or DMSO (*n* = 3) for 48 h. **C** Western blots results and relative protein quantification of H2AK9ac, H3K4ac, H3K9ac, H3K14ac, H3K18ac, H3K23ac, H4k5ac, H4K8ac, H4K12ac, HDAC1, HDAC2, HDAC3, HDAC4 and HDAC6 after treated by BRD4770 5 μM or DMSO for 48 h in RAVSMCs, and total protein served as a loading control (*n* = 4), *p < 0.05. **D** Cell images and histogram of RAVSMCs infected with lenti-HDAC1, lenti-flag, and then treated with BRD4770 5 μM or DMSO (*n* = 3) for 48 h. **E** Cell images and histogram of RAVSMCs infected with lenti-HDAC3, lenti-flag, and then treated with BRD4770 5 μM or DMSO (*n* = 3) for 48 h. **F** Cell images and histogram of RAVSMCs incubated LMK235 1 μM for 2 h before treated with BRD4770 5 μM or DMSO (*n* = 3) for 48 h. **G** Cell images and histogram of RAVSMCs incubated Tubastain A 2.5 μM for 2 h before treated with BRD4770 5 μM or DMSO (*n* = 3) for 48 h. Mark “ns” means no statistical sense

As demonstrated previously, EHMT2 was irresponsible for the inhibitory effect of BRD4770 against VSMCs proliferation. There might be one another mysterious molecule that mediated the anti-proliferative effect. Serum response factor (SRF), a widely expressed transcription factor in all cell types, was considered in the first place. It was reported that SRF was required for not only the expression of many SMC differentiation marker genes, but also for the initial differentiation of SMC during development [19]. In physiological environment, SRF promoted the proliferation or differentiation of VSMCs [20]. Besides, it was also showed that SRF stabilized SKP2-containing ubiquitin complex level, regulated immediate early genes, and formed SRF/myocardin axis with its main cofactor, myocardin, all of which participated in the modulation of VSMCs proliferation [21]. In the present work, a decrease in SRF level was found after BRD4770 treatment (Fig. 6A). Therefore, a SRF over-expressed VSMC line was constructed for further study. However, results showed that the cell number suppressed by BRD4770 was not rescued under over-expressed SRF (Fig. 6B), indicating that SRF was not involved in the cell proliferation and regulation of BRD4770. According to the recent studies, an association between histone acetylation/deacetylation and VSMCs proliferation [22] has been proposed. The acetylation of histone in VSMCs was evaluated by western blot test. Results showed that the expression of acetylated histones like H2AK9ac, H3K4ac, H3K9ac, H3K14ac, H3K18ac, H3K23ac, H4k5ac, H4K8ac and H4K12ac were down regulated by BRD4770. Moreover, the quantity of histone deacetylases, including HDAC1, HDAC2 and HDAC3 decreased, while HDAC4 and HDAC6 increased (Fig. 6C). It has been observed that the knockdown of HDAC1 and HDAC3 decreased SMC proliferation and neointima formation in murine models of vascular injury [22]. However, in the present work, results showed that the overexpression of HDAC1 and HDAC3 didn't increase the cell number that suppressed by BRD4770 in our research (Fig. 6D and E). Meanwhile, it was demonstrated that interfering HDAC4 inhibited the proliferation of VSMCs [23]. As to HDAC6, tubastatin A, an inhibitor of HDAC6, downregulated platelet-derived growth factor–induced VSMC proliferation and migration, while HDAC6 overexpression exerted the opposite effect [24]. Thus, we treated VSMCs with HDAC4 inhibitor, LMK235 [25], and HDAC6 inhibitor, tubastatin A, for indicated time before BRD4770 incubation. Results showed that the inhibition of HDAC4 or HDAC6 did not reverse cell numbers that suppressed by BRD4770 (Fig. 6F and G). All taken together, although SRF and histone acetylation were found to be altered after the treatment of BRD4770, the cell number suppression by BRD4770 was neither via SRF nor HDACs, which required to be further explored.

### BRD4770 regulated cellular histone methylation and SUV39H2 overexpression reversed the reduction of cell number

**Fig. 7:**
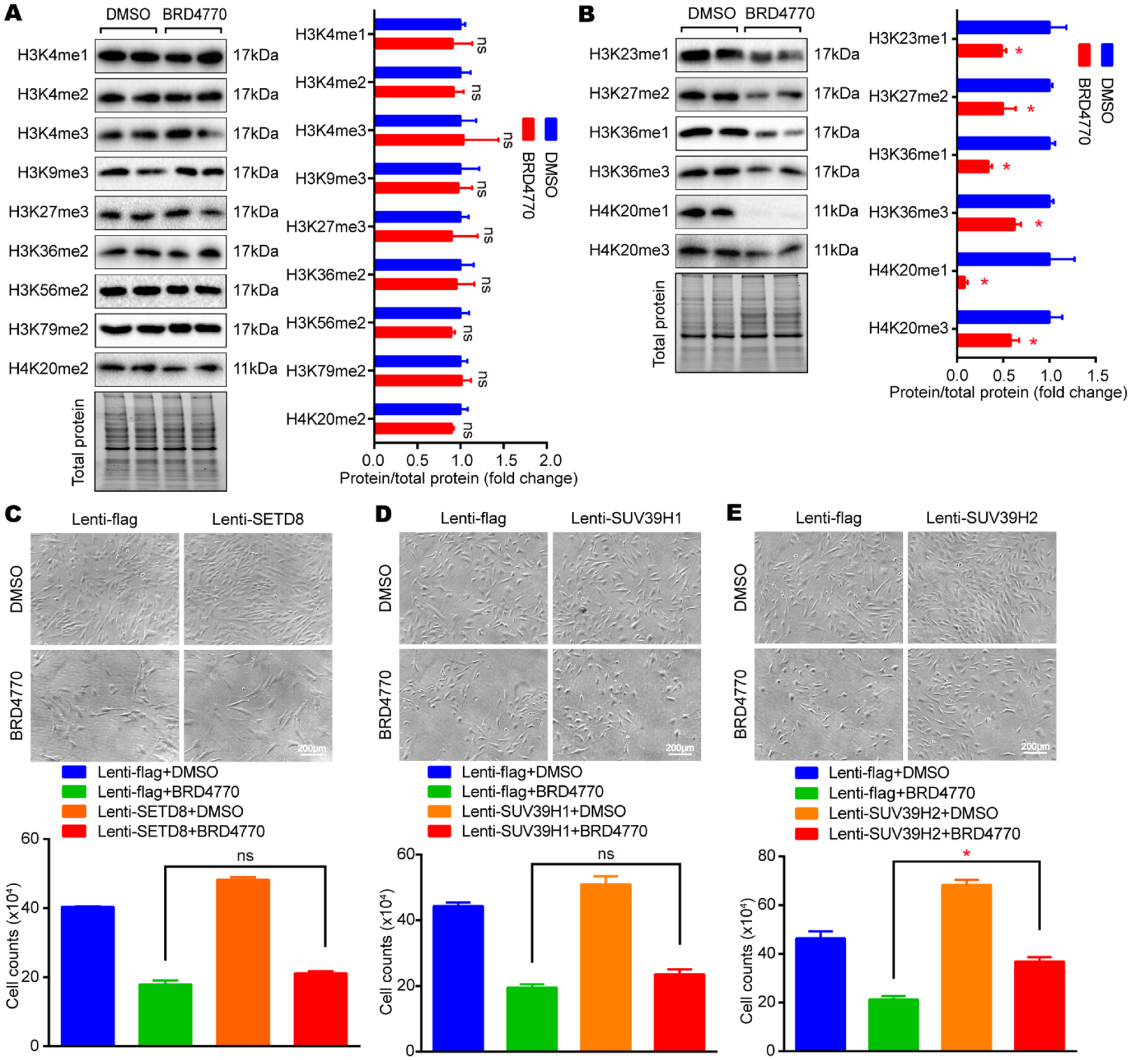
SUV39H2 rescued VSMCs number that inhibited by BRD4770. **A** Western blots results and relative protein quantification of H3K4me1, H3K4me2, H3K4me3, H3K9me3, H3K27me3, H3K36me2, H3K56me2, H3K79me2 and H4K20me2 after treatment of BRD4770 5 μM or DMSO for 48 h in RAVSMCs, total protein served as a loading control (*n* = 4). **B** Western blots results and relative protein quantification of H3K23me1, H3K27me2, H3K36me1, H3K36me3, H4K20me1 and H4K20me3 after treatment of BRD4770 5 μM or DMSO for 48 h in RAVSMCs, total protein served as a loading control (*n* = 4), *p < 0.05.** C** Cell images and histogram of RAVSMCs infected with lenti-SETD8, lenti-flag, and treated with BRD4770 5 μM or DMSO (*n* = 3) for 48 h. **D** Cell images and histogram of RAVSMCs infected with lenti-SUV39H1, lenti-flag, and treated with BRD4770 5 μM or DMSO (*n* = 3) for 48 h.** E** Cell images and histogram of p-HAVSMCs infected with lenti-SUV39H2, lenti-flag, and treated with BRD4770 5 μM or DMSO (*n* = 3) for 48 h, *p < 0.05. Mark “ns” means no statistical sense

The methylation of histone was also studied by western blot. Results showed that the expression of H3K4me1, H3K4me2, H3K4me3, H3K9me3, H3K27me3, H3K36me2, H3K56me2, H3K79me2 and H4K20me2 had no noticeable difference before/after BRD4770 treatment (Fig. 7A), while H3K23me1, H3K27me2, H3K36me1, H3K36me3, H4K20me1 and H4K20me3 remarkably decreased after the treatment of BRD4770 in VSMCs (Fig. 7B).

As showed in Fig. 7B, it is showed that the expression of H4K20me1 was significantly inhibited by BRD4770. Meanwhile, KMT5A/SETD8, the upstream histone transmethylase of H4K20me1, was demonstrated to catalyze monomethylation of H4K20, which eventually promoted chromatin compaction [26]. Moreover, to investigate the relation between BRD4770 and SETD8, one of the vital regulators of G2/M checkpoint, SETD8 over expressed in VSMCs. We found that the suppressed cell number induced by BRD4770 was not reversed by the overexpression of SETD8 (Fig. 7C). Considering the H3K9 dimethylation (H3K9me2) and H3K9 trimethylation (H3K9me3) [27], suppressor of variegation 3–9 homolog 1 (SUV39H1) and suppressor of variegation 3–9 homolog 2 (SUV39H2) were taken into account. Both SUV39H1 and SUV39H2 were reported to catalyze the formation of H3K9me2 and H3K9me3, and also participated the cell cycle regulation in a variety of cells, including the epidermal stem and progenitor cells [28] and lung cancer cells [29]. As noted in the preceding text, H3K9me1/2 was inhibited by BRD4770 (Fig. 1A), SUV39H1/2 was evaluated, considering that SUV39H1/2 was a transmethylase that specifically trimethylates H3K9 using H3K9me1 as its substrate. The SUV39H1/2 overexpressed VSMCs was constructed, and found that SUV39H1 overexpression didn't rescue cell number (Fig. 7D). However, it was exciting to found that the cell number inhibited by BRD4770 was noticeably rescued when SUV39H2 over expressed (Fig. 7E). To sum up, results indicated that histone methylation participated in the proliferative regulation of VSMCs induced by BRD4770, especially SUV39H2 might be the key transmethylase involved.

### *BRD4770 suppressed neointima formation of injured vessels *in vivo

**Fig. 8:**
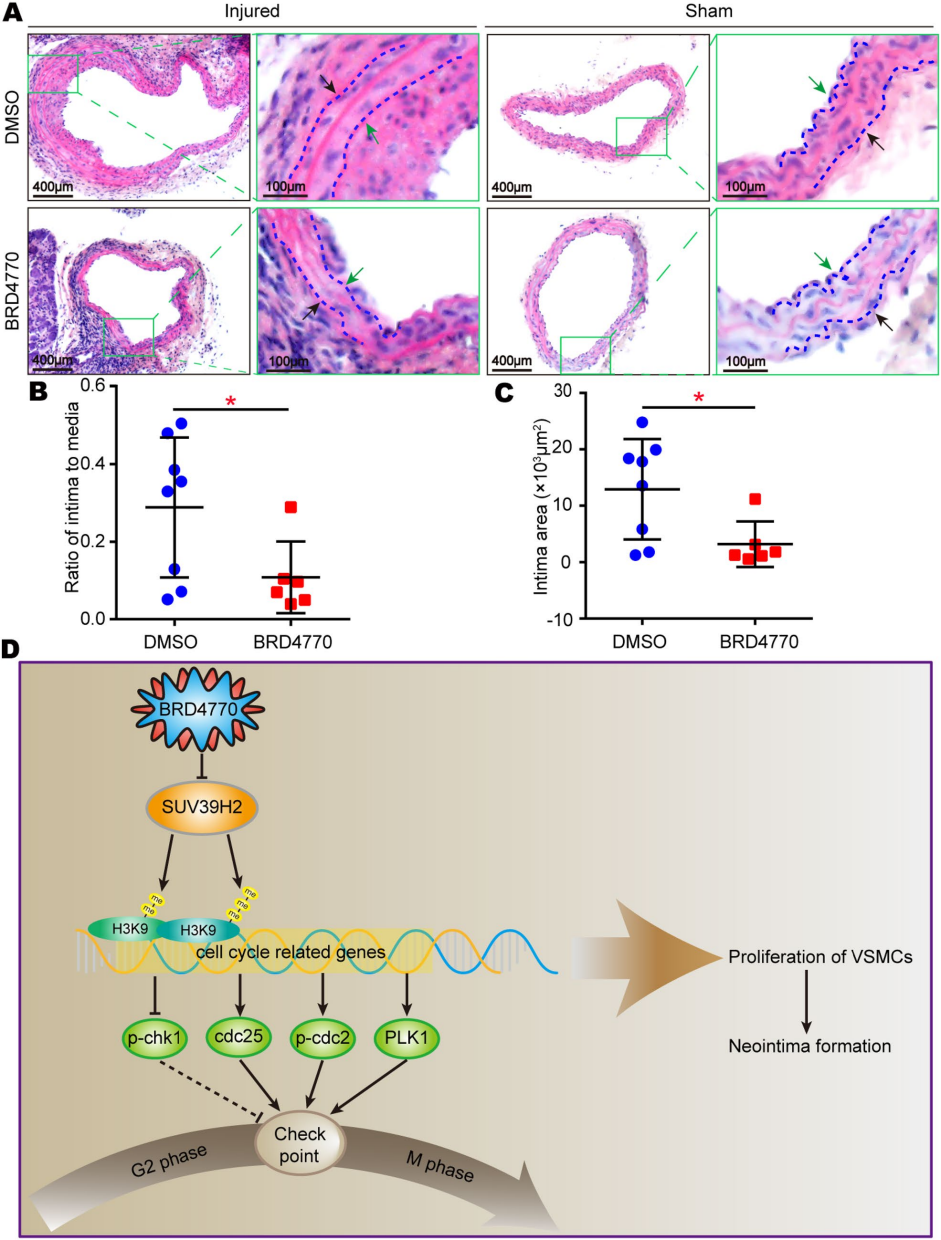
BRD4770 inhibited neointima formation of injured vessels. **A** HE-stained sections showed the structure of carotid arteries of DMSO and BRD4770 treated mice at 28th day post-injury. The insets in the left panels were magnified, and the magnified images are presented in the right panels. The black arrows indicated external elastic discs and green arrows indicated inner elastic discs. **B** The intima/media ratios were quantified (*n* = 6–8), *p < 0.05. **C** The intimal areas were quantified (*n* = 6–8), *p < 0.05. **D** Schematic summary of total article

To verify the effects after BRD4770 treatment in vivo, mice carotid artery wire injury surgery was performed. It was showed that the thickness of newly formed neointima got significantly thinner after BRD4770 treated. Moreover, both the ratio of intima to media and the area of intima in BRD4770 treated arteries were smaller than control group (Fig. 8A–C). Collectively, it was indicated that BRD4770 suppressed neointima formation in vessels both in vivo and in vitro.

Altogether, results in the present work suggested that BRD4770 regulated the expression of cell cycle related proteins, induced G2/M phase blockage and inhibited the proliferation of VSMCs, which subsequently inhibited neointima formation in vessels in vivo. And the SUV39H2 might be functioned as the key protein to regulate all these processes (Fig. 8D).

## Discussion

The proliferation of VSMCs is involved in the process of neointima formation. We have previously reported that the inhibition of transmethylase EHMT2 induced the autophagy of VSMCs [14]. Despite BRD4770 was a known compound that inhibited the expression of EHMT2, our data revealed that BRD4770 exhibited an inhibitory effect on VSMCs with a novel mechanism which was independent on autophagy and EHMT2. It was showed that BRD4770 blocked cell cycle in G2/M phase and inhibited cell proliferation via suppressing SUV39H2. Furthermore, BRD4770 ameliorated vascular intimal hyperplasia in vivo.

BRD4770 was reported to affect the growth of carcinoma cells in recent years. In pancreatic cancer, BRD4770 induced senescent phenotype and activated the ATM (ataxia telangiectasia mutated) pathway to induce DNA damage without affecting ATR (ATM- and Rad3-related) pathway, which ultimately restrained the cell growth [16]. However, the impact of BRD4770 on VSMCs was rarely reported. Our previous study revealed the relationship between EHMT2 and autophagy in VSMCs. In the present study, BRD4770, a robust inhibitor of EHMT2, inhibited cell growth independently on EHMT2 and autophagy, which suggested that VSMCs phenotypic switch mediated by BRD4770 did not related with EHMT2 or autophagy. Furthermore, flow cytometry test revealed that cell number in G2/M phase was increased markedly while cell number in G1/S phase was relatively decreased after BRD4770 treatment, which indicated that cells were blocked in G2/M phase. Additionally, our data indicated that the expression of proteins and mRNA of G2/M phase was affected by BRD4770. Till now, the findings suggested that VSMCs proliferation inhibition by BRD4770 might due to the cell cycle blockage in G2/M phase. Urrutia et al. showed BRD4770 affected cell growth, DNA synthesis, cell cycle progression at S phase, and DNA-damage signaling, after combination with Checkpoint kinase1(Chk1) inhibitor prexasertib in pancreatic cancer [30]. In liver cancer, BRD4770 induced the expression of pyruvate dehydrogenase kinase 4 (PDK4) by epigenetic regulation of PDK4 related H3K9me2/3 [15]. Moreover, BRD4770 combined with gossypol was reported to increase LC3-II levels and the autophagosome number in PANC-1 cells [31] based on their previous research findings that BRD4770 could induce senescence in PANC-1 cells [16]. In our study, we found that BRD4770 inhibited proliferation of cultured VSMCs and suppressed VIH in injured carotid arteries. The cell cycle blockage in G2/M phase by BRD4770 mediated anti-proliferative effect in VSMCs.

SUV39H2(KMT1B), a critical histone methyltransferase, was a member of SUV39 sub-family of KMTs that regulated histone H3K9 di-/tri-methylation, transcriptional regulation and cell cycle in cycling and non-cycling cells [32, 33]. In reviewing the literature, SUV39H2 functioned to control cellular behavior by restricting diverse genes expression through H3K9me3 repressive effect which anchored with target gene promoters. For example, Balmer reported that SUV39H2 controlled fate conversion of epidermal stem and progenitor cells by restricting conversion through H3K9me3 repressive marks on gene promoters encoding components of the Wnt/p63/adhesion axis [28]. Meanwhile, some other research group verified that SUV39H2 could also affect cell behavior by non-methyltransferase action. SUV39H2 directly binded to the SLIT1 promoter, suppressing SLIT1 transcription to enhance proliferation and metastasis in colorectal cancer [34]. Moreover, prior studies also noted that SUV39H2 was highly expressed in glioma tissues and the knockdown of SUV39H2 induced the inhibition of proliferation, stemness and cell growth in glioma cell[35]. And the depletion of SUV39H2 caused an increase in the population of G1 phase and induced apoptosis in osteosarcoma [36]. Totally, published results showed that SUV39H2 could regulate cell behavior through H3K9 or direct regulation, and SUV39H2 played a vital role in cell life. However, the role of SUV39H2 in the cardiovascular system, especially in VSMCs, was still rarely reported. In our study, we demonstrated that SUV39H2 instead of G9a reversed the cell number inhibition induced by BRD4770, which indicated that BRD4770 inhibited VSMCs proliferation via SUV39H2 instead of G9a.

Previous studies noted that the proliferation and migration of VSMC was the important progression of VIH. It was also evidenced that the epigenetic modifications also participated in neointimal hyperplasia with restenosis [37]. Consistent with in vitro study, BRD4770 significantly ameliorated restenosis of injured carotid arteries observed in mice. Additionally, we found that BRD4770 affected the expression of cell cycle related genes besides G2/M phase through transcriptomic analysis and RT-PCR test. The reason might be effects on variable epigenetic regulation of histones as Figs. 6 and 7 demonstrated. Furthermore, we found the expression of H3K9me3 did not significantly changed after BRD4770 treatment. In general, it seems that BRD4770 might affect some other histone transmethylases which reversely regulated H3K9me3 concurrently. Therefore, the impact of BRD4770 on VSMCs was complicated and detailed pathway needed further research.

In conclusion, BRD4770 showed an excellent suppressive effect in VSMCs proliferation. BRD4770 might be a new compound against the proliferation of VSMCs, while the effect of BRD4770 on endotheliocytes or other organs remained unknown. Still, further research should be focused on the dosage and the delivery methods of BRD4770.

## Conclusions

Our results indicated that BRD4770 blocked cell cycle in G2/M phase and inhibited VSMCs proliferation via SUV39H2 instead of EHMT2 or autophagy in VSMCs. The treatment of BRD4770 was a benefit to protect against restenosis. Pharmacologic manipulation of BRD4770 may be a potential therapeutics for VIH, such as vascular restenosis.

## Supplementary Information

Below is the link to the electronic supplementary material.

Supplementary file1 (DOCX 18 kb)

## Data Availability

The data that support the findings of this study are available from the corresponding author upon reasonable request.

## Abbreviations

CAD: Coronary artery disease
VIH: Vascular intimal hyperplasia
SVG: Saphenous vein grafts
VSMCs: Vascular smooth muscle cells
SUV39H: Suppressor of variegation 3–9 homolog
HDAC: Histone deacetylase
SRF: Serum response factor
p-HAVSMCs: Primary human aortic vascular smooth muscle cells
RAVSMCs: Rabbit aortic vascular smooth muscle cells
LDH: Lactate dehydrogenase
RT-PCR: Real-time polymerase chain reaction
FPKM: Fragments per kilobase of exon per million fragments mapped
DEGs: Differentially expressed genes
KEGG: Kyoto Encyclopedia of Genes and Genomes
CC: Cell component
BP: Biological process
GSEA: Gene set enrichment analysis
p-chk1: Phosphorylated checkpoint kinase1
p-cdc2: Phosphorylated checkpoint kinase 2
PLK1: Polo like kinase 1
CDC: Cell division cycle
Chk1: Checkpoint kinase1
PDK4: Pyruvate dehydrogenase kinase 4
ATM pathway: Ataxia telangiectasia mutated pathway
ATR pathway: ATM- and Rad3-related pathway
