# Supplementary material for: BRD4770 inhibits vascular smooth muscle cell proliferation via SUV39H2, but not EHMT2 to protect against neointima formation
Source: Hum Cell. 2023 Jun 12;36(5):1672–88. doi: 10.1007/s13577-023-00924-4 (PMC10390615; doi:10.1007/s13577-023-00924-4)
Supplement: Supplementary file 1 — Supplementary file1 (DOCX 18 kb) [file 13577_2023_924_MOESM1_ESM.docx]

**Supplementary Table S1. Primers for plasmid construction.**

| **Primers** | **Sequence(5’-3’)** |
| --- | --- |
| **Primers for overexpression** |  |
| EHMT2-F | CCGACGCGTGCCACCATGCGGGGTCTACCGAGAG |
| EHMT2-R | ACGCGTCGACTGTGTTGACAGGGGGCAGG |
| SETD8-F | CCGACGCGTGCCACCATGGCTAGAGGCAGGAAGATGTC |
| SETD8-R | ACGCGTCGACATGCTTCAGCCACGGGTG |
| HDAC1-F | GCCACGCGTGCCACCATGGCGCAGACGCAGG |
| HDAC1-R | ATCCTCGAGGGCCAACTTGACCTCCTCCT |
| HDAC3-F | GCCACGCGTGCCACCATGGCCAAGACCGTGGCCTATTTCTACG |
| HDAC3-R | ATCCTCGAGAATCTCCACATCGCTTTCCTTGTC |
| SUV39H1-F | CCGACGCGTGCCACCATGGCGGAAAATTTAAAAGGCTG |
| SUV39H1-R | CCGCTCGAGGAAGAGGTATTTGCGGCAG |
| SUV39H2-F | CGACGCGTGCCACCATGGCGGCGGTCGG |
| SUV39H2-R | CCGCTCGAGGTTGAGGTAACCTCTGCAAGTC |
| SRF-F | CCGACGCGTGCCACCATGTTACCGACCCAAGCTGG |
| SRF-R | CCGCTCGAGTTCACTCTTGGTGCTGTGGG |
| **Primers for knock down** |  |
| shSQSTM1-F | CCGGCCGAATCTACATTAAAGAGAACTCGAGTTCTCTTTAATGTAGATTCGGTTTTTG |
| shSQSTM1-R | AATTCAAAAACCGAATCTACATTAAAGAGAACTCGAGTTCTCTTTAATGTAGATTCGG |
| shBECN1-F | CCGGCCCGTGGAATGGAATGAGATTCTCGAGAATCTCATTCCATTCCACGGGTTTTTG |
| shBECN1-R | AATTCAAAAACCCGTGGAATGGAATGAGATTCTCGAGAATCTCATTCCATTCCACGGG |

**Supplementary Table S2.** Primary antibodies for Western blotting assay**.**

| **Name** | **Item No.** | **Corp.** |
| --- | --- | --- |
| LC3II/I | #12741 | Cell Signaling Technology |
| p-Chk1(Ser345)(133D3) | #2348 | Cell Signaling Technology |
| p-cdc2(Tyr15)(10A11) | #4539 | Cell Signaling Technology |
| SRF | #5147S | Cell Signaling Technology |
| HDAC1 | #5356 | Cell Signaling Technology |
| HDAC2 | #5113 | Cell Signaling Technology |
| HDAC3 | #3949 | Cell Signaling Technology |
| HDAC4 | #7628 | Cell Signaling Technology |
| HDAC6 | #7558 | Cell Signaling Technology |
| H3K14ac | #7627 | Cell Signaling Technology |
| H3K4me2 | #9725 | Cell Signaling Technology |
| H3K4me3 | #9727 | Cell Signaling Technology |
| H3K27me2 | #9728 | Cell Signaling Technology |
| H3K27me3 | #9733 | Cell Signaling Technology |
| H3K36me1 | #14111 | Cell Signaling Technology |
| H3K79me2 | #5427 | Cell Signaling Technology |
| p62 | ab56416 | Abcam |
| Beclin1 | ab55878 | Abcam |
| H3 | ab1791 | Abcam |
| H2AK9ac | ab177312 | Abcam |
| H3K4ac | ab176799 | Abcam |
| H3K18ac | ab40888 | Abcam |
| H3K23ac | ab61234 | Abcam |
| H4K5ac | ab51997 | Abcam |
| H4K8ac | ab15823 | Abcam |
| H4K12ac | ab46983 | Abcam |
| H3K4me1 | ab8895 | Abcam |
| H3K9me1 | ab9045 | Abcam |
| H3K9me2 | ab1220 | Abcam |
| H3K23me1 | ab176132 | Abcam |
| H3K36me2 | ab9049 | Abcam |
| H3K36me3 | ab9050 | Abcam |
| H3K56me2 | ab177294 | Abcam |
| H4K20me1 | ab9051 | Abcam |
| H4K20me3 | ab9053 | Abcam |
| p-H3 | sc-8656-R | Santa Cruz |
| PCNA | GTX100539 | Genetex |
| H3K9ac | GTX630554 | Genetex |
| H3K9me3 | GTX121677 | Genetex |
| H4K20me2 | GTX54112 | Genetex |

**Supplementary Table S3. Primers for Real-Time PCR detection.**

| **Primers** | **Sequence(5’-3’)** |
| --- | --- |
| **Primers for human** |  |
| hum-PLK1-F | TGACTCAACACGCCTCATCC |
| hum-PLK1-R | GCTCGCTCATGTAATTGCGG |
| hum-CCNB1-F | GAAACGCATTCTCTGCGACC |
| hum-CCNB1-R | ACACCCAGCAGAAACCAACA |
| **Primers for rabbit** |  |
| rab-PLK1-F | GCTTCCACGGCTTTTTCGAG |
| rab-PLK1-R | GCCAGCCCAAAATCCCCTAT |
| rab-CCNB1-F | ATCTGTGTCAGGCGTTCTCG |
| rab-CCNB1-R | TCCCGACCCAGGAGGTATTT |
| rab-CDC25B-F | GCATGTGCCGTTTCATCAGG |
| rab-CDC25B-R | AGAACTCCTTGTAGCCCCCT |
| rab-CDC25C-F | CACCCCCTCCAAAATATCGGT |
| rab-CDC25C-R | ACCAAGGAAAAGGCGAAACC |
| rab-CDK2-F | CCTTTGGAGTGCCTGTTCGT |
| rab-CDK2-R | CTAGATAGGAGCACAGCGGG |
| rab-PIM1-F | GACGCTTGCTCTGTTTGTGG |
| rab-PIM1-R | AGGCACACCATCCAGAATCG |
| rab-CDKN1A-F | CAGGTAGCACAGCCCAGAAG |
| rab-CDKN1A-R | AGCCGCTACTCAAAGTGCAA |
| rab-CDKN2A-F | GACTTCAGGGGTGTCACGTTC |
| rab-CDKN2A-R | CTCTTTCTTCCTCTGGGGCTG |
| rab-CDKN2B-F | CAAACGTGGAGCGGCACTAA |
| rab-CDKN2B-R | CTTAATCGGGCAGGAAGCCC |
| rab-CDKN2C-F | AAATGGGGGCGGGTTTTTCA |
| rab-CDKN2C-R | TAGCCGGTCCCCTAAAATCG |
| rab-ORC1-F | ACAGGTCAGCAATACTGGCA |
| rab-ORC1-R | TTGTCTGGTCTTCAGCCTTGT |
| rab-ATM-F | TGCTCTTTTGGACAGTGGGG |
| rab-ATM-R | CGGCTGGTTTGTGAGTTGTG |
| rab-MCM2-F | TCGCTCGCTATCTGTCGTTC |
| rab-MCM2-R | TTTCGCTTCAGGTCATGGCT |
| rab-MCM3-F | AGTGCTGGACGATGTGGAACT |
| rab-MCM3-R | TTCCCTGGTCTTCCTCGTCAT |
| rab-MCM4-F | AAGGGCAAAACACCAGCTCT |
| rab-MCM4-R | TCGCAGGGCTTCTTCAAACA |
| rab-MCM6-F | CCCGTGTCAGTGGTGTTGAT |
| rab-MCM6-R | CAGGCAAGAAAGACCAGCCT |
| rab-CDC20-F | GCAATTTCCAGCGCACTACA |
| rab-CDC20-R | GTGCAGGTCACTCTCGAACA |
| rab-KIF18B-F | CTCCTGATGGTGCCTTGGTT |
| rab-KIF18B-R | ACAGACGCGCTGAAGTACAA |
